# Association Between the Remnant Cholesterol Inflammation Index and Cardiac Syndrome X

**DOI:** 10.3390/diagnostics16081113

**Published:** 2026-04-08

**Authors:** İbrahim Aktaş, Erdoğan Yaşar, Kadir Uçkaç

**Affiliations:** 1 Department of Cardiology, Faculty of Medicine, Malatya Turgut Özal University, 44090 Malatya, Türkiye; ibrahim.aktas@ozal.edu.tr (İ.A.); drkadiruckac@gmail.com (K.U.); 2 Department of Cardiology, Malatya Training and Research Hospital, 44090 Malatya, Türkiye

**Keywords:** microvascular dysfunction, remnant cholesterol inflammation index (RCII), Cardiac Syndrome X

## Abstract

**Background and Objectives:** Cardiac Syndrome X (CSX), a clinical entity within the Ischaemia with Non-Obstructive Coronary Arteries (INOCA) spectrum, is increasingly recognised as an inflammatory and systemic vascular disorder. Remnant cholesterol (RC) and inflammation are emerging contributors to residual cardiovascular risk; however, their combined role in microvascular angina remains unclear. This study aimed to evaluate the association between the remnant cholesterol inflammation index (RCII), integrating RC and high-sensitivity C-reactive protein (hs-CRP), and the clinical presence of CSX. **Methods:** This single-centre, retrospective observational study included 392 individuals who underwent coronary angiography between January 2023 and January 2025. The study population comprised 197 patients diagnosed with CSX and 195 control subjects with normal coronary anatomy and no objective evidence of myocardial ischaemia. RC was calculated as total cholesterol minus the sum of LDL-C and HDL-C, and RCII was derived as RC × hs-CRP. Importantly, invasive microvascular testing (e.g., CFR or IMR) was not performed. Logistic regression analyses were performed to identify independent predictors of CSX, and receiver operating characteristic (ROC) curve analysis was used to evaluate diagnostic performance. **Results:** Patients with CSX exhibited significantly higher levels of hs-CRP, SII, and RCII compared with controls (all *p* < 0.001). In the multivariable logistic regression analysis, RCII demonstrated an independent association with CSX (odds ratio 1.095, 95% confidence interval 1.060–1.131; *p* < 0.001). ROC curve analysis showed that RCII provided moderate but significant discrimination for CSX (area under the curve [AUC] 0.765, 95% CI 0.695–0.795). Pairwise comparisons confirmed that RCII had a significantly higher AUC than RC, hs-CRP, or SII individually. **Conclusions:** Higher RCII levels appear to be significantly associated with the clinical diagnosis of CSX. By integrating atherogenic remnant cholesterol burden and systemic inflammation, RCII may serve as a valuable composite biomarker for identifying residual inflammatory lipid risk. Rather than acting as a definitive diagnostic tool, these findings warrant further validation in large-scale prospective cohort studies.

## 1. Introduction

Cardiac Syndrome X (CSX) is a clinical condition characterised by typical angina pectoris symptoms triggered by exercise and objective evidence of myocardial ischaemia on stress tests, despite coronary angiography revealing normal epicardial coronary arteries or arteries with non-obstructive narrowing [1]. Up to approximately 40% of patients presenting with stable angina symptoms and undergoing coronary angiography may have completely normal epicardial coronary arteries. After ruling out the possibility of coronary vasospasm, this clinical picture is termed CSX and is associated with microvascular dysfunction [2,3]. Today, this condition is evaluated under the heading of “Ischaemia with Non-Obstructive Coronary Arteries” (INOCA), and it is accepted that the underlying cause of the symptoms is coronary microvascular dysfunction [4]. Current data indicate that these patients have reduced quality of life and an increased risk of major adverse cardiovascular events (MACE) compared to healthy individuals [5]. It is becoming increasingly clear that CSX is not merely a functional disorder but a systemic vascular disease closely associated with inflammation, endothelial dysfunction, and oxidative stress [6]. It has been demonstrated that low-grade inflammation impairs endothelial function by reducing nitric oxide bioavailability, which in turn increases susceptibility to ischaemia by disrupting microvascular tone. Comprehensive meta-analyses and clinical studies have also indicated that inflammatory markers (NLR, CRP, SII, etc.) are significantly elevated in CSX patients compared to healthy controls, suggesting that inflammation plays a decisive role in both the onset and progression of CSX [7,8,9].

Traditionally, low-density lipoprotein (LDL) cholesterol has been the primary focus in cardiovascular risk management. However, despite achieving LDL targets, a residual cardiovascular risk persists in many patients. In this context, remnant cholesterol (RC), which is the cholesterol content of triglyceride-rich lipoproteins (such as VLDL and IDL), emerges as a causal risk factor in the atherosclerotic process, independent of LDL [10]. RC can penetrate the arterial wall without requiring oxidation, accumulate in the intima, and exacerbate endothelial dysfunction by triggering low-grade inflammation in the vascular wall [11]. This synergistic relationship between remnant cholesterol and systemic inflammation can be better defined using novel composite biomarkers such as the “Remnant Cholesterol Inflammation Index” (RCII). Recent studies have shown that high RCII levels predict the risk of myocardial infarction, stroke, and all-cause mortality more strongly than each factor (RC or CRP) alone [12,13,14]. RC’s potential to initiate endothelial dysfunction and exacerbate vascular inflammation suggests that it may play a key role not only in macrovascular but also in microvascular angina pathogenesis.

Several cell-based inflammatory indices (e.g., NLR, SII) lack the lipid-driven component of atherogenesis; RCII bridges this gap by integrating both metabolic (remnant cholesterol) and inflammatory (hs-CRP) triggers. This dual integration makes it potentially more tailored to the pathophysiology of microvascular angina. Although its impact on macrovascular events is known [13,15], the relationship between RCII and microvascular dysfunction remains unclear. Therefore, this study aimed to evaluate the association between RCII and CSX, investigating its potential as a valuable biomarker for identifying residual inflammatory lipid risk in this population.

## 2. Materials and Methods

### 2.1. Study Design, Population and Grouping

This study was designed as a single-centre, retrospective observational study examining the data of patients who presented to the cardiology clinic of Malatya Training and Research Hospital between 15 January 2023 and 15 January 2025 with typical chest pain or angina-equivalent symptoms and underwent coronary angiography (CAG) due to suspected ischaemic heart disease.

A total of 510 patients were initially included in the study. Of these patients, 118 were excluded because they did not meet the established inclusion criteria. The study group comprised 197 patients who had objective evidence of myocardial ischaemia (typical angina precipitated by exercise; transient ischaemic ST-segment depressions of ≥1 mm, bradycardia or hypotension occurring during the treadmill exercise test; reversible perfusion abnormalities on exercise myocardial perfusion scintigraphy) but normal coronary arteries on coronary angiography and were diagnosed with Cardiac Syndrome X (CSX). The control group comprised 195 individuals with normal coronary anatomy who were referred to our centre for coronary angiography due to atypical angina symptoms, had low-risk exercise test results, and no objective evidence of ischaemia.

Patients with the following conditions were excluded from the study:

1. History of previous myocardial infarction (MI), percutaneous coronary intervention (PCI) or coronary artery bypass grafting (CABG) (12 patients).
2. Detection of visible plaque, luminal irregularity, muscular bridge, or ectasia in any major epicardial artery on angiography (57 patients).
3. Systolic heart failure (left ventricular ejection fraction < 40%) (8 patients).
4. Moderate or severe valvular disease (6 patients).
5. Active infection, malignancy, or known systemic inflammatory/autoimmune disease (due to potential impact on CRP levels) (18 patients).
6. Severe renal (eGFR < 30 mL/min/1.73 m^2^) or hepatic insufficiency (2 patients).
7. Use of systemic steroids or immunosuppressive therapy.
8. Patients taking lipid-lowering medication within the last 3 months (15 patients).

**Figure 1:**
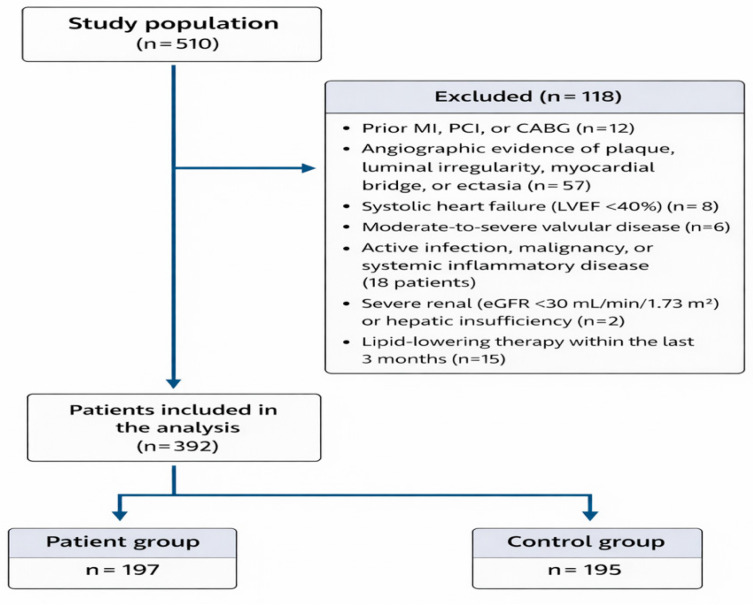
Flowchart of study population selection and group allocation.

The study flow is shown in Figure 1.

The patients’ demographic characteristics (age, gender), cardiovascular risk factors (hypertension, diabetes, smoking, dyslipidaemia) and medication history were obtained from the hospital records system. Hypertension was defined according to the ESH/ESC hypertension guidelines (≥140/90 mmHg or use of antihypertensive treatment); diabetes was defined according to the ADA criteria (previously established diagnosis, medication use, or fasting plasma glucose ≥ 126 mg/dL) accepted [16,17].

### 2.2. Blood Sample Collection and Processing

Venous blood samples were collected on the morning of the procedure following at least 12 h of overnight fasting. Complete blood count, biochemical parameters (glucose, creatinine, serum albumin) and lipid profile [total cholesterol (TC), high-density lipoprotein cholesterol (HDL-C), triglycerides (TG)] were measured using fully automated analysers. LDL-C was calculated using the Friedewald formula. Direct LDL values were used in patients with high triglyceride levels or where direct LDL measurement was available. High-sensitivity C-reactive protein (hs-CRP) levels were measured by immunoturbidimetric method (mg/L). Remnant Cholesterol (RC): RC (mg/dL) = Total Cholesterol − (LDL-C + HDL-C). The Remnant Cholesterol Inflammation Index, the main independent variable of this study, was calculated using the formula: RCII = RC (mg/dL) × hs-CRP (mg/L) [18,19]. Additionally, for comparison purposes, the Systemic Immune-Inflammation Index (SII) was calculated using the following formula: SII = (Platelets × Neutrophils)/Lymphocytes. In addition, other inflammatory indices such as the neutrophil/lymphocyte ratio (NLR) and platelet/lymphocyte ratio (PLR) were also calculated from the complete blood count.

### 2.3. Echocardiographic Assessment

All patients underwent standard two-dimensional transthoracic echocardiography prior to angiography. Left ventricular ejection fraction (LVEF) was calculated using Simpson’s two-plane method; valve morphology and gradients were assessed to exclude moderate and severe valve disease. Those with LVEF < 40% were excluded from the study.

### 2.4. Coronary Angiography and Assessment of Microvascular Dysfunction

Coronary angiography was performed via the femoral or radial route using the standard Judkins technique. The left anterior descending (LAD), circumflex (LCx) and right coronary artery (RCA) were visualised in at least two projections. Vasodilators were not routinely used during the procedure, and a hyperventilation test was performed to rule out coronary vasospasm. A normal coronary anatomy was defined as coronary angiographic images demonstrating entirely smooth vessel contours without any observable stenosis or luminal irregularity. To assess coronary microvascular function, the Myocardial Blush Grade (MBG) was evaluated for each major coronary artery. MBG was scored from 0 (no myocardial blush) to 3 (normal myocardial blush). The Total Myocardial Blush Score (TMBS) was calculated by summing the grades of the left anterior descending, left circumflex, and right coronary arteries, with a maximum possible score of 9.

### 2.5. Statistical Analysis

Statistical analyses were performed using SPSS 26.0 (IBM Corp., Armonk, NY, USA) and MedCalc Statistical Software version 23.2 (MedCalc Software Ltd., Ostend, Belgium). The distribution of continuous variables was assessed using the Kolmogorov–Smirnov test. Data showing a normal distribution were presented as mean ± standard deviation, while data not normally distributed were presented as median (minimum–maximum). Categorical variables were expressed as percentages (%). For comparisons between two groups, Student’s *t*-test was used for normally distributed data, and the Mann–Whitney U test was used for non-normally distributed data. Categorical variables were analysed using the chi-square (χ^2^) test or Fisher’s exact test when appropriate. Spearman’s correlation analysis was used to evaluate the association between MBG and RCII. Univariate and multivariate logistic regression analysis was performed to determine the independent predictors of microvascular dysfunction. Variables with a *p* < 0.05 significance level in the univariate analysis were included in the model. To prevent multicollinearity, two separate multivariable logistic regression models were constructed: Model 1 evaluated the individual components (remnant cholesterol and hs-CRP), whereas Model 2 incorporated the composite RCII. Additionally, variance inflation factors (VIF) were calculated for Model 2 to rule out collinearity between the co-included composite indices. The VIF values for both RCII and SII were strictly <2.0, confirming the absence of significant multicollinearity. For interpretability, RCII was additionally standardized using z-score transformation, and standardized effect estimates were reported alongside the original scale. ROC curve analysis was performed to determine the optimal cut-off value for RCII; the area under the curve (AUC) and the corresponding 95% confidence intervals were reported. A *p* < 0.05 value was considered statistically significant in all analyses. Pairwise comparisons between ROC curves were performed using the DeLong test to evaluate whether differences in AUC values were statistically significant.

## 3. Results

A total of 392 participants were enrolled in the study, comprising 197 patients diagnosed with CSX and 195 control subjects. The baseline demographic, clinical and laboratory characteristics of the study population are summarized in Table 1 and Table 2.

There were no significant differences between the two groups in terms of age (60.1 ± 11.0 vs. 60.7 ± 8.7 years, *p* = 0.732), gender distribution (*p* = 0.843), hypertension, smoking status, and left ventricular ejection fraction (*p* > 0.05 for all). However, the prevalence of diabetes mellitus was significantly higher in the patient group compared to the control group (39.5% vs. 22.0%, *p* = 0.016). TMBS and MBG were lower in the patient group. Regarding lipid profiles, Total Cholesterol (191 ± 41 vs. 182 ± 32 mg/dL, *p* = 0.045) and Low-Density Lipoprotein (LDL) cholesterol (119 ± 38 vs. 110 ± 32 mg/dL, *p* = 0.035) levels were significantly higher in the patient group. Spearman correlation analysis demonstrated a significant inverse relationship between RCII levels and TMBS (r = −0.61, *p* < 0.001). More notably, inflammatory markers showed marked elevation in CSX patients. High-sensitivity C-reactive protein (hs-CRP) levels were significantly higher in the patient group compared to controls (3.9 ± 1.8 vs. 1.8 ± 0.6 mg/L, *p* < 0.001). Similarly, the median SII was significantly elevated in the patient group (789 [IQR: 395–2301] vs. 468 [IQR: 255–1517], *p* < 0.001). Consistent with these findings, RCII was found to be significantly higher in patients with CSX compared to the control group.

Univariate and multivariate logistic regression analyses were performed to identify independent predictors of CSX (Table 3).

In the univariate analysis, diabetes mellitus (OR = 1.252, *p* = 0.042), remnant cholesterol (OR = 1.213, *p* = 0.020), hs-CRP (OR = 3.204, *p* < 0.001), SII (OR = 1.012, *p* < 0.001), and the composite index RCII (OR = 1.390, *p* = 0.001) were significantly associated with CSX. Traditional factors such as age, sex, and hypertension did not reach statistical significance in this cohort.

To avoid mathematical coupling between the composite index and its individual components, multivariable evaluation was conducted using two separate models. In Model 1, which included diabetes alongside the individual lipid and inflammatory markers, both remnant cholesterol (OR = 1.187, 95% CI: 1.015–1.390, *p* = 0.045) and hs-CRP (OR = 2.152, 95% CI: 1.251–3.657, *p* = 0.001) remained independent factors associated with CSX. In Model 2, the individual components were replaced by the composite index (RCII). After adjustment, RCII was independently associated with CSX (OR: 1.095, 95% CI: 1.060–1.131, *p* < 0.001). When standardized, each 1-standard deviation increase in RCII was associated with higher odds of CSX (adjusted OR: 2.35, 95% CI: 1.73–3.19, *p* < 0.001). In this model, SII remained statistically significant (OR = 1.002, *p* = 0.035), whereas diabetes mellitus lost its independent significance (OR = 0.908, *p* = 0.341). Furthermore, variance inflation factor (VIF) analysis for Model 2 confirmed the absence of multicollinearity between the co-included inflammatory indices, yielding VIF values strictly <2.0 for both RCII and SII.

**Figure 2:**
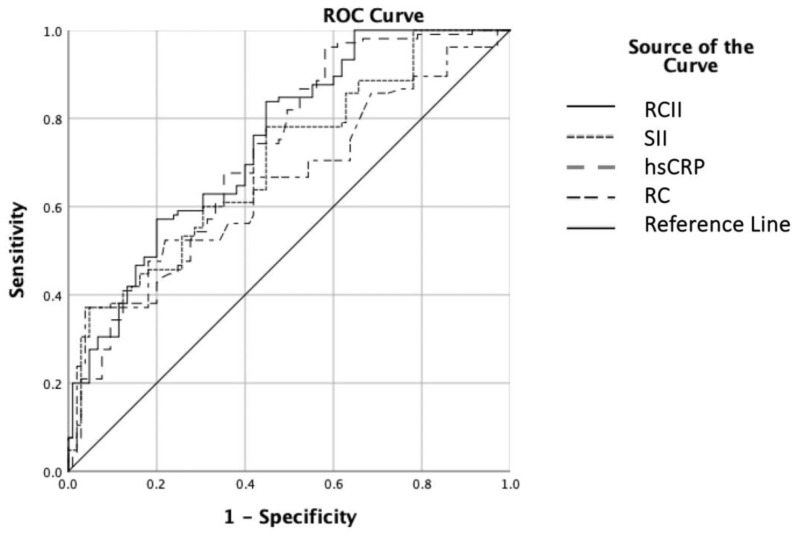
Receiver operating characteristic (ROC) curves of RCII, SII, hs-CRP, and RC for predicting microvascular dysfunction in CSX patients. hs-CRP, high-sensitivity C-reactive protein; RC, remnant cholesterol; RCII, remnant cholesterol inflammation index; SII, systemic immune-inflammation index.

Receiver operating characteristic (ROC) curve analysis showed that RCII was significantly associated with CSX, with an area under the curve (AUC) of 0.765 (95% CI: 0.695–0.795; *p* < 0.001) and a sensitivity of 76% and specificity of 69% at a cut-off value of >9.9. The AUC values for the other parameters were 0.695 (95% CI: 0.640–0.750; *p* < 0.001) for SII, 0.680 (95% CI: 0.600–0.760; *p* < 0.001) for hs-CRP, and 0.650 (95% CI: 0.585–0.695; *p* < 0.001) for remnant cholesterol. Figure 2.

Importantly, pairwise comparisons of the ROC curves using the DeLong test revealed that the AUC of RCII (0.765) was significantly higher than the AUCs of remnant cholesterol (0.650, *p* = 0.020), hs-CRP (0.680, *p* = 0.027), and SII (0.695, *p* = 0.045).

## 4. Discussion

This study is one of the first to investigate the diagnostic and predictive value of the Remnant Cholesterol Inflammation Index (RCII), a novel biomarker in patients with Cardiac Syndrome X. The main finding of our study is that RCII levels were significantly higher in the patient group with microvascular dysfunction compared to the control group. These findings suggest that the synergistic interaction of atherogenic lipid load and systemic inflammation may play a role not only in macrovascular atherosclerosis but also in microvascular pathology.

Endothelial dysfunction, autonomic dysregulation, and particularly chronic low-grade inflammation are known to play a central role in the pathophysiology of Cardiac Syndrome X [1,20]. Previous studies have shown that inflammatory markers such as CRP, NLR, and Systemic Immune-Inflammation Index (SII) are elevated in CSX patients and that this elevation is associated with impaired coronary flow reserve [9,21,22]. The observed association between RCII and microvascular dysfunction in our study suggests that microvascular damage may not be solely related to inflammation but could also be linked to metabolic lipid burden.

Recent reviews emphasise that coronary microvascular dysfunction (CMD) is not a condition limited to the coronary circulation but shares common pathomechanisms with the microvascular circulations of the brain, kidneys, and other organs as a cardiac reflection of systemic microvascular disease [23,24]. In contrast to non-obstructive coronary anatomy, which is classically considered “benign,” CMD is now well known to be associated with an increased risk of major adverse cardiovascular events (MACE); it also significantly impairs patients’ quality of life. Therefore, the early identification of high-risk phenotypes at the microvascular level is critical not only for symptom management but also for improving long-term prognosis. Our study demonstrates an independent association between RCII and microvascular dysfunction, suggesting that, upon prospective validation, this index may aid in risk stratification for the INOCA/CSX spectrum.

The relationship between microvascular dysfunction and inflammation has previously been demonstrated in patient groups with non-obstructive coronary anatomy using various haematological indices. Yaşar et al. reported that the SII (platelet × neutrophil/lymphocyte ratio) was significantly elevated in patients with CSX and independently predicted microvascular dysfunction [9]. Balta et al. showed that NLR in CSX patients increased in parallel with both normal coronary artery controls and carotid intima-media thickness, favouring endothelial damage and subclinical inflammation [22]. These data support the concept that microvascular ischemia is characterized by an inflammatory phenotype. Our findings suggest that RC represents the metabolic/lipoprotein component of this inflammatory phenotype and that RCII may capture a more “atherogenic” burden in relation to microvascular damage than haematological indices such as SII and NLR.

Remnant cholesterol is the cholesterol content of triglyceride-rich lipoproteins and has the ability to penetrate the arterial wall independently of LDL and without requiring oxidative modification, and to be directly taken up by macrophages to form foam cells [25]. There is strong evidence in the literature that RC increases the risk of acute coronary syndrome, stroke, and all-cause mortality [26,27]. However, data on the effect of RC on the microvascular bed are limited. Gao et al. reported that high RC levels are associated with poor prognosis in patients with MINOCA [28]. This suggests that RC may cause ischaemia by inducing plaque erosion or endothelial dysfunction at the microvascular level, even in the absence of epicardial stenosis. Similarly, elevated RC levels in patients with chronic total occlusion (CTO) have been shown to impair coronary collateralisation, a microvascular adaptation [29]. Our results support that these adverse effects of RC are also valid in patients with stable microvascular angina and that RC may contribute to symptoms by impairing endothelium-dependent vasodilation.

Numerous studies have demonstrated that hs-CRP levels are a marker of both epicardial atherosclerosis and systemic inflammatory risk, with higher hs-CRP levels independently associated with plaque burden, plaque vulnerability, and long-term event risk [30,31]. Recent experimental and clinical data indicate that the CRP and IL-6 axis is associated with endothelial dysfunction, reduced NO bioavailability, and impaired microvascular tone regulation, reinforcing the concept of inflammation-related CMD [32]. In this context, RCII—defined as the product of RC and hs-CRP—integrates both lipoprotein-mediated vascular burden and inflammatory activity into a single parameter, may provide a useful tool for the assessment of CMD.

The superiority of RCII over RC or CRP alone as a biomarker is primarily attributable to the “vicious cycle” between dysregulated lipid metabolism and inflammation. Genetic and observational studies have demonstrated that elevated RC levels trigger low-grade inflammation in the vascular wall [33]. Free fatty acids and monoacylglycerols released through the hydrolysis of TRLs activate the local inflammatory response, thereby exacerbating endothelial damage. Therefore, RCII combines both the metabolic burden initiating the pathology (RC) and the vascular response to it (CRP) into a single index, reflecting the underlying pathophysiology of microvascular dysfunction more comprehensively. Chen et al. demonstrated that the effect of RC and CRP on stroke risk occurs via reciprocal mediation and that the two factors together pose a stronger risk than either alone [15]. Zhang et al. reported that synergistic assessment of remnant cholesterol and CRP in coronary artery disease patients better predicted moderate-to-high SYNTAX score risk compared to the separate use of either RC or CRP alone [34]. This parallels the findings of Yu et al. regarding the prognostic superiority of RCII in ischaemic stroke prognosis [35]. Our study extends this line of reasoning from the epicardial level to the microvascular level and provides a unique contribution by demonstrating that the combined effect of RC and inflammation is also associated with microvascular dysfunction in the CSX/INOCA spectrum.

In clinical practice, the standard lipid panel (LDL-C) may often be within normal limits in CSX patients, which may lead to patients being misclassified as “low risk” and not receiving adequate medical treatment. Our study demonstrates that even if LDL-C targets are achieved, high RCII levels may indicate a persistent “residual risk”. The fact that RCII can be readily calculated from routine blood tests provides a cost-effective, supplementary tool for identifying high-risk patients. Furthermore, elevated RCII levels may help define a distinct patient phenotype that could be considered for inclusion in future clinical trials evaluating targeted anti-inflammatory or TRL-lowering strategies.

From a clinical perspective, both residual cholesterol burden and residual inflammatory risk are defined as a sequential “dual target” in current guidelines and reviews. In various cohorts, it has been demonstrated that individuals with high remnant cholesterol levels have a significantly higher incidence of myocardial infarction, atherosclerotic events, and coronary lesion severity [36,37]. Similarly, residual inflammatory risk, as defined by hs-CRP, emerges as an independent predictor of long-term event risk even under modern statin therapy [30]. A composite index such as RCII has the potential to go beyond classical LDL-focused risk measures by capturing both dimensions simultaneously. Particularly in symptomatic CSX patients with well-controlled LDL levels, a high RCII may reveal a “residual atherogenic-inflammatory burden” and provide an argument for more aggressive lifestyle or pharmacological interventions.

Although invasive functional assessments like CFR or IMR were not performed in our study, we utilized the Total Myocardial Blush Score (TMBS) as an objective angiographic surrogate for microvascular function [38]. The significantly lower TMBS observed in the patient group provides supportive evidence of impaired microvascular function.

Our study has some limitations. First, the retrospective, cross-sectional, and single-centre design precludes the establishment of a definitive causal relationship (i.e., whether elevated RCII directly leads to microvascular dysfunction or vice versa) and may introduce selection bias. Due to ethical constraints related to invasive angiography, the control group included symptomatic rather than truly healthy individuals, potentially attenuating the observed associations. Second, the diagnosis of microvascular dysfunction was based on clinical criteria and non-invasive ischemia testing; invasive confirmation via CFR, IMR, or provocative acetylcholine testing was not performed. Third, residual confounding cannot be excluded, as key metabolic parameters (e.g., insulin resistance and menopausal status) and detailed echocardiographic data were not systematically recorded in this retrospective registry; therefore, these variables were not included in the analysis. Finally, the lack of long-term follow-up data precludes evaluation of the prognostic impact of RCII. Future large-scale, prospective studies incorporating invasive functional tests are warranted to better define the prognostic value of RCII in CSX patients.

## 5. Conclusions

Higher RCII levels are significantly associated with the presence of CSX. By reflecting both atherogenic lipid burden and systemic inflammatory activity, RCII may serve as a simple and accessible composite biomarker for identifying residual inflammatory lipid risk in this complex patient population. Rather than acting as a definitive diagnostic tool, it offers a practical approach to evaluating the intertwined metabolic and inflammatory pathways underlying microvascular angina. However, given the cross-sectional nature of this study, large-scale prospective cohort studies are required to clarify potential causal relationships and validate its clinical utility.

## Figures and Tables

**Table 1:** Clinical, Demographic and Angiographic Characteristics of Groups.

	Control Group (n = 195)	Patient Group (n = 197)	*p*
Age (years)	60.7 ± 8.7	60.1 ± 11.0	0.732
Female sex, n (%)	105 (53.8)	102 (51.7)	0.843
Diabetes mellitus, n (%)	43 (22)	78 (39.5)	0.016
Ejection Fraction, (%)	60.1 ± 8.0	59.8 ± 5.0	0.942
Hypertension, n (%)	59 (30.2)	64 (32.4)	0.812
Systolic BP (mmHg)	124.7 ± 15.2	125.2 ± 14.4	0.696
Diastolic BP (mmHg)	79.6 ± 8.4	80.3 ± 7.5	0.951
Smoking, n (%)	39 (20)	46 (23.3)	0.486
BMI, kg/m^2^	23.8 ± 2.2	24.1 ± 1.9	0.360
Beta-blocker use, n (%)	21 (10.7)	31 (15.7)	0.147
ACE-inhibitor/ARB use, n (%)	26 (13.3)	29 (14.7)	0.690
TMBS	8.6 ± 0.8	7.5 ± 1	<0.001
MBG LAD	2.9 ± 0.6	2.6 ± 0.4	0.045
MBG LCx	2.8 ± 0.4	2.4 ± 0.5	0.025
MBG RCA	2.9 ± 0.1	2.5 ± 0.6	0.033

ACE, Angiotensin-Converting Enzyme; ARB, Angiotensin Receptor Blocker; BMI, Body Mass Index; BP, Blood Pressure; LAD, Left Anterior Descending Artery; LCx, Left Circumflex Artery; MBG, Myocardial Blush Grade; RCA, Right Coronary Artery; TMBS, Total Myocardial Blush Score.

**Table 2:** Laboratory Parameters of Groups.

	Control Group (n = 195)	Patient Group (n = 197)	*p*
Glucose (mg/dL)	117.1 ± 53	139 ± 61	0.060
Total cholesterol (mg/dL)	182 ± 32	191 ± 41	0.045
Low density lipoprotein cholesterol (mg/dL)	110 ± 32	119 ± 38	0.035
High density lipoprotein cholesterol (mg/dL)	54 ± 13	43 ± 10	<0.001
Triglycerides (mg/dL)	115 ± 45	195 ± 78	<0.001
Hemoglobin, g/dL	13.7 ± 1.3	13.6 ± 1.9	0.793
Neutrophil count, 10^3^/μL	5.2 ± 1.2	5.8 ± 1.4	0.012
Platelets count, 10^3^/μL	215 ± 71	249 ± 42	0.001
Lymphocyte count, 10^3^/μL	2.1 ± 0.5	2.0 ± 0.6	0.029
NLR	2.1 ± 0.5	3.4 ± 1.2	<0.001
PLR	108 ± 22	131 ± 30	<0.001
Remnant cholesterol (mg/dL)	18 ± 7	29 ± 14	<0.001
RCII	5.1 ± 3.4	14.8 ± 9.4	<0.001
hs-CRP (mg/L)	1.8 ± 0.6	3.9 ± 1.8	<0.001
SII (median) [IQR] (×10^3^)	468 (255–1517)	789 (395–2301)	<0.001

hs-CRP, High-sensitivity C-Reactive Protein; NLR, Neutrophil/Lymphocyte Ratio; PLR, Platelet/Lymphocyte Ratio; RCII, Remnant Cholesterol Inflammation Index; SII, Systemic Immune Inflammation Index.

**Table 3:** Univariate and Multivariate Analysis for Prediction of CSX.

Variables	Univariate Analysis OR (95% CI)	*p*-Value	Multivariable Model 1 OR (95% CI)	*p*-Value	Multivariable Model 2 OR (95% CI)	*p*-Value
Female sex	1.146 (0.748–1.675)	0.779	–	–	–	–
Age	0.975 (0.902–1.012)	0.284	–	–	–	–
Hypertension	1.139 (0.712–1.887)	0.834	–	–	–	–
Diabetes mellitus	1.252 (1.080–1.452)	0.042	1.010 (0.739–1.382)	0.078	0.908 (0.712–1.180)	0.341
Remnant cholesterol	1.213 (1.021–1.407)	0.020	1.187 (1.015–1.390)	0.045	–	–
hs-CRP	3.204 (2.189–6.503)	<0.001	2.152 (1.251–3.657)	0.001	–	–
SII	1.012 (1.010–1.015)	<0.001	1.003 (1.001–1.006)	0.001	1.002 (1.001–1.005)	0.035
RCII	1.390 (1.110–1.603)	0.001	–	–	1.095 (1.060–1.131)	<0.001

CRP, C-reactive protein; hs-CRP, High-sensitivity C-Reactive Protein; RCII, Remnant Cholesterol Inflammation Index; SII, Systemic Immune Inflammation Index.

## Data Availability

The data presented in this study are available on request from the corresponding author due to privacy.

## Abbreviations

The following abbreviations are used in this manuscript:

CFR	Coronary flow reserve
IMR	Index of microcirculatory resistance
RCII	Remnant cholesterol inflammation index
SII	Systemic immune-inflammation index
